# Thermosensitive porphyrin-incorporated hydrogel with four-arm PEG-PCL copolymer (II): doxorubicin loaded hydrogel as a dual fluorescent drug delivery system for simultaneous imaging tracking *in vivo*

**DOI:** 10.1080/10717544.2017.1289570

**Published:** 2017-03-10

**Authors:** Xia Dong, Hongli Chen, Jingwen Qin, Chang Wei, Jie Liang, Tianjun Liu, Deling Kong, Feng Lv

**Affiliations:** 1 Tianjin Key Laboratory of Biomedical Materials, Institute of Biomedical Engineering, Chinese Academy of Medical Sciences & Peking Union Medical College, Tianjin, PR China and; 2 School of Life Science and Technology, Xinxiang Medical University, Xinxiang, Henan, PR China

**Keywords:** Imaging tracking, multispectral fluorescence, drug delivery, porphyrin, hydrogel

## Abstract

Visualization of a drug delivery system could reveal the pharmacokinetic properties, which is essential for the design of a novel drug delivery system. *In vivo* optical imaging offers an advanced tool to monitor the drug release process and the therapeutic effect by the combination of fluorescence imaging and bioluminescence imaging. Multispectral fluorescence imaging can separate the drug and the carrier without interference. Herein, a dual fluorescent anti-tumor drug delivery system was monitored with the doxorubicin-loaded hydrogel to further explore the application of the porphyrin-incorporated hydrogel with four-arm PEG-PCL copolymer as a drug carrier, based on the beneficial fluorescence and good biocompatibility of the porphyrin incorporated hydrogel. Using nude mice bearing luciferase expressed hepatic tumor as models, the whole process from the drug delivery to the tumor therapeutic effects were real time visualized simultaneously after administration at interval from 0 to 18 d. The imaging results suggest that the fluorescence signals of the drug and the carrier can be separated and unmixed from the drug-loaded hydrogel successfully, avoiding the interference of the fluorescence signals. The tumor growth or inhibition can be real time tracked and analyzed quantitatively by bioluminescence imaging. Noninvasive continuous tracking the *in vivo* drug delivery process simultaneously is a potential trend for the precise drug delivery and treatment.

## Introduction

It is crucial to reveal the pharmacokinetic properties of a drug delivery system for the translational medicine. A detailed knowledge of the drug delivery system including the drug release and the carrier degradation is essential for the design of a novel drug delivery system. The release and distribution of the drug, and the location and degradation of the carrier *in vivo* are closely related to the therapeutic effect. Generally, an *in vitro* or *ex vivo* evaluation is commonly performed to investigate the drug delivery process. The *in vivo* drug release, distribution and metabolism were evaluated by high-performance liquid chromatography (HPLC) or radioactivity measurement in the blood samples at intervals or the anatomical based tissue samples (Greenaway et al., 2010; Woods et al., 2015). The carrier degradation and metabolism can be assessed by other *in vitro* or *ex vivo* physicochemical assays such as the gravimetric, volume, molecular weight, mechanical properties, morphology and viscosity determination of periodic samples except of the above methods (Bruggeman et al., 2008; Liang et al., 2011; Undin et al., 2014). The main limitations of these methods rely on the invasion and destruction for the samples in addition to the sample quantity requirements because of the lack of real-time *in vivo* tracking objectively. Noninvasive continuous tracking is a potential trend for the investigation of the *in vivo* drug delivery process simultaneously (Tzu-Yin et al., 2013; Brudno et al., 2014; Lin et al., 2016; Zhu et al., 2016).

With the increasing development of the medical imaging equipments, methods and skills, the *in vivo* imaging monitoring has been explored to the assessment of the drug delivery system (Appel et al., 2013; Ahmed Abdelbary et al., 2015; Zhou et al., 2015; Xia et al., 2016). Imaging visualization can enable specific location and targeting drug delivery in the diseased tissue. Among these medical imaging techniques, optical imaging technique is advantageous because of its high sensitivity, low radiation, non-invasion and long-term monitoring (Hilderbrand & Weissleder, 2010; Sevick-Muraca, 2012). *In vivo* optical imaging has been successfully applied in the fields of the tumor and inflammation diagnosis, the monitoring of tissue repair therapy and the drug delivery tracking (Selvam et al., 2011; Zhang et al., 2011; Wohl-Bruhn et al., 2012; Zhang et al., 2016). In the field of noninvasive tracking for the drug delivery, optical imaging can monitor the drug release and distribution, the location and degradation of the carrier, and the therapeutic effect simultaneously by the combination of fluorescence imaging and bioluminescence imaging, which is unique and advanced compared to other imaging methods (Chen et al., 2015; Ma et al., 2015). Fluorescence imaging can be serviced for the multispectral analysis of two or more fluorescent signals from the drug delivery system by the separation of fluorescent drug and carrier (Zhou & El-Deiry, 2009; Hoffmann et al., 2012; Dong et al., 2016b). For instance, a dual fluorescent drug delivery system based on N-(2-hydroxypropyl)methacrylamide (HPMA) copolymer was monitored simultaneously for passive tumor targeting with pH-sensitive drug release *in vivo* (Hoffmann et al., 2012). In our previous reports, several dual fluorescent drug loaded hydrogel systems have been successfully tracked with multispectral fluorescence imaging by subcutaneous injections (Dong et al., 2015; Dong et al., 2016b). Multispectral fluorescence imaging can respectively locate and monitor the drug and the carrier without any interferes in the optimal imaging condition. The choice of the fluorescent drug and the design of the fluorescent carrier play a vital and decisive role for the tracking of the drug delivery system.

Fluorescent drug is the basis of the fluorescence imaging tracking. In the tracking of the drug release, fluorescent drugs include self-fluorescence emission drugs and exogenous fluorescent tags labeled drugs (Etrych et al., 2016; Winzen et al., 2016). Self-fluorescence emission drugs can stand for the actual status of the drug delivery objectively compared to the fluorescent model drugs or exogenous fluorescent tags labeled drugs. Doxorubicin (Dox) is an anti-tumor drug with the intense self-fluorescence in a wide range of the spectrum, attracting more attention in the fluorescence tracking of the drug delivery system for the tumor therapy (Li et al., 2013; Kruger et al., 2014; Dong et al., 2017). Fluorescent carrier is an another concern for the tracking of the drug delivery system (Ghaderi et al., 2011; Zhang & Yang, 2013). Fluorescent carriers tend to be labeled by the incorporation or the conjugation at the end of the inorganic and organic fluorescent dyes (Cunha-Reis et al., 2013; Wang et al., 2014). The major disadvantages of these methods include the weak biocompatibility and the poor stability, because the fluorescent tags can be broken away from the polymer, preventing the continuous tracking for the polymer and hampering the biotissue with the penetration of the free molecule or particle. In our previous reports, fluorescent copolymer was designed to overcome their deficiency using fluorescent porphyrin compounds as the backbone and core of the copolymer (Lv et al., 2014; Dong et al., 2016b). Porphyrin is a special fluorescent compound as a component of hemoglobin, which assigns it favorable biocompatibility beyond other fluorescent dyes (Rieffel et al., 2015; Huang et al., 2016). The porphyrin core in the polymer backbone not only ensures continuous fluorescence tracking efficiency by avoiding the early selective breakage of the ectogenic fluorescent tag, but also decreases the adverse effect to the biotissue from the separated fluorescent dyes. The fluorescence imaging results *in vivo* demonstrated that the porphyrin incorporated hydrogel with four-arm PEG-PCL copolymer (POR-PEG-PCL) has the beneficial fluorescence and good biocompatibility as a hydrogel implant or a nanogel probe (Lv et al., 2014; Dong et al., 2016c), suggesting its enormous potential for the image tracking.

In this article, a dual fluorescent anti-tumor drug delivery system was monitored with the Dox loaded porphyrin conjugated hydrogel to further explore the application of the POR-PEG-PCL hydrogel as a drug carrier. Although other fluorescent drug delivery systems have been investigated using rhodamine as the model drug by subcutaneous injections in our report (Dong et al., 2016b), it is necessary that the porphyrin incorporated hydrogel based drug delivery system with therapeutic effects was further tracked by an intratumoral implantation with significant clinical potential due to the micro environment difference from the tumor and the subcutaneous tissue. Using nude mice bearing luciferase expressed hepatic tumor as models, the whole process from the drug delivery to the tumor therapeutic effects can be visualized by bioluminescence imaging and multispectral fluorescence imaging. Moreover, the interrelation of the drug release and distribution, the location and degradation of the carrier, and the tumor growth or inhibition can be further illustrated according to the real time imaging location and tracking. Visualization of the drug delivery system could be offer a new approach for the accurate administration.

## Experimental section

### Materials

POR-PEG-PCL copolymer was ring-opening copolymerized with ɛ-CL and porphyrin-conjugated PEG using stannous octoate as catalyst, as reported previously (Lv et al., 2014). Dox was provided from Dakub Meilun biology technology Co., Ltd. (Dalian, PR China). Chloral hydrate (>99.0, pharmaceutical grade) was purchased from Yulong Algae Co., Ltd. (Qingdao, PR China). D-luciferin potassium salt was obtained from Gold Biotechnology, Inc. (Olivette, MO). Other reagents were all analytic reagent (AR) grade.

Balb/c nude mice (seven weeks old, 20–25 g) were performed for the *in vivo* imaging, housed in cages with free access to food and water. All animal procedures were conducted following the protocol approved by the Institutional Laboratory Animal Ethics Committee and the Institutional Animal Care and Use Committee (IACUC), Peking Union Medical College, PR China. All animal experiments were performed in compliance with the Guiding Principles for the Care and Use of Laboratory Animals, Peking Union Medical College, PR China.

### Sol–gel–sol phase transition, thermal analysis, drug release and fluorescence imaging *in vitro*

The sol–gel–sol phase transition photos of the hydrogel and the Dox loaded hydrogel were taken with a concentration of 40% from 10 to 60 C with a heating rate of 1 °C min^−1^. The gel and sol status were defined as “no flow” and “flow” in 1 min using the tube-inversion method. The vials were imaged to record the gel and sol status at a temperature of 20, 37 and 50 °C, respectively.

Differential scanning calorimetry (DSC) (Q2000, TA instruments, New Castle, DE) was used to analyze the thermal properties of the Dox and the Dox loaded POR-PEG-PCL copolymer at a temperature range from 0 to 250 °C under a nitrogen atmosphere at a heating and cooling rate of 5 °C min^−1^.

The *in vitro* drug release curve of the Dox from the hydrogel was performed by a multimode microplate spectrum photometer (Varioskan TM Flash, ThermoFisher Scientific, Waltham, MA) in PBS of pH 5.5 or 7.4 at 37 °C. At preset time points, the extra fluid was taken for the Dox analysis with 2 mL by absorbance spectrum and was followed by the addition of the same volume of fresh PBS. The concentration of the Dox was calculated from a standard curve of known Dox absorbance of 485 nm. All experiments were performed in triplicate.

After gelation, the Dox loaded hydrogel was taken for multispectral fluorescence imaging *in vitro* to separate the carrier and the drug with dual excitation wavelengths of 523 and 595 nm by an *in vivo* imaging system (Maestro EX, CRi Inc., Woburn, MA).

### Multispectral fluorescence imaging tracking of the Dox loaded hydrogel and bioluminescence imaging monitoring for the tumor therapy

Balb/c nude mice bearing hepatic tumor were modeled for imaging monitoring of the drug delivery and the tumor therapy with fluorescence imaging and bioluminescence imaging, respectively. Luciferase expressed hepatic cells Bel-7402 (1 × 10^6^) in 0.1 mL of normal saline (NS) were subcutaneously injected into the armpit region of nude mice to model the mice bearing hepatic tumor. When the tumor volume reached approximately 100 mm^3^, the nude mice were randomly assigned to the experimental groups and control group (*n* = 6 for each group). Free Dox, the hydrogel and the Dox loaded hydrogel were intratumoral injected with the same concentration and dose of 20 mg/kg Dox, respectively. At predetermined time points, the mice were anesthetized by an intraperitoneal injection of chloral hydrate for imaging tracking.

The drug delivery was tracked with multispectral fluorescence imaging by the Maestro CRI *in vivo* imaging system with dual excitation wavelengths of 523 and 595 nm at an exposure time of 300 ms. The imaging tracking of the drug release and the materials erosion were performed after administration at interval from 0 to 18 d. The single signal of the Dox and the hydrogel can be separated with green and red by the spectral species unmixing from the cube file. The quantitative analysis of the fluorescence signals of the drug and the hydrogel was carried out by the Maestro software.

The inhibition and growth of the tumor was imaging monitored by bioluminescence imaging at interval for each three days from 0 to 18 d. Following the intraperitoneal injection with D-luciferin potassium salt solution for 5 min, the bioluminescence imaging was carried out by *in vivo* imaging system (IVIS Lumina system, Xenogen Corporation, Alameda, CA). In addition, the tumor region was irradiated by near-infrared (NIR) laser at power density of 0.5 W cm^−2^ for 20 min each time every day for the irradiation groups. Then the tumor inhibition rate was quantitatively calculated by the imaging software.

## Results and discussion

### Sol–gel–sol phase transition and drug loading

**Figure 1.**
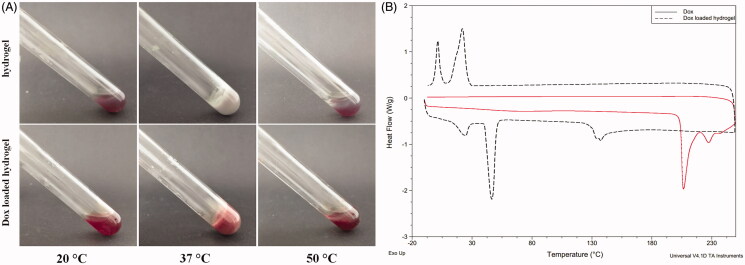
Sol–gel–sol phase transition and thermal properties of the Dox loaded hydrogel ((A). Photograph of a sol state at 20 °C, a gel state at 37 °C and a precipitate at 50 °C; (B) DSC curve of the Dox and the Dox loaded porphyrin copolymers at a temperature range from 0 to 250 °C).

Hydrogels are paid much attention as drug delivery deposits or tissue engineering scaffolds (Song et al., 2015; Zhu et al., 2015). The injectability of the hydrogel based drug delivery system is a basic requirement to facilitate the delivery from a sol state at room temperature to a gel state at body temperature. Since POR-PEG-PCL hydrogel is a thermosensitive hydrogel, the drug loaded POR-PEG-PCL hydrogel should remain its beneficial injectability. The sol–gel–sol transition of the hydrogel and the drug-loaded hydrogel is presented in Figure 1(A). Just as the POR-PEG-PCL hydrogel, the drug loaded hydrogel can also undergoes a sol–gel–sol phase transition as the temperature increases, transforming into a non-flowing gel at physiological temperature from an injectable flowing sol at room temperature. The red drug loaded hydrogel formed due to the embedment of the Dox while the POR-PEG-PCL hydrogel is light purple with the characteristic signal of the porphyrin compound. With the further increasing of the temperature, the non-flowing gel turns into a precipitate with the gel–sol phase transition. The hydrogel state can locate to the targeted region until it degrades gradually at body temperature as the body temperature is lower than the gel–sol phase transition temperature, which ensures the sustained drug delivery of the hydrogel.

In order to investigate the loading effect of the Dox to the hydrogel, differential scanning calorimetry (DSC) was performed to assess the thermal properties of the drug-loaded hydrogel. As shown in Figure 1(B), the Dox has a melting peak at 205 °C, suggesting its crystalline state of the free Dox. When the Dox is loaded into the copolymer, the melting peak of the Dox disappeared, because the Dox was entrapped into the nano micells of the copolymer and then formed the hydrogel as a result of the effective loading of the drug instead of the physical mixing. Besides, the Dox loaded POR-PEG-PCL copolymer exhibited the exothermic peaks and the crystallization peaks of PEG and PCL segments, respectively. The loading of the drug did not generate obvious change to the thermal properties of the copolymer.

### Drug release *in vitro*

**Figure 2.**
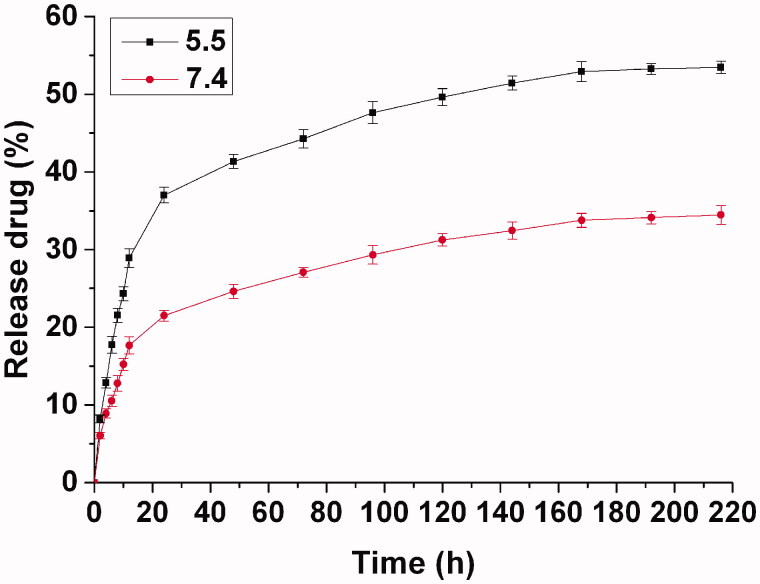
Drug release from hydrogel in vitro in PBS at pH of 5.5 or 7.4. The concentration of released Dox was calculated from a standard curve of known absorption of 485 nm. The results were expressed as mean ± SD (*n* = 3).

The Dox loaded hydrogel was prepared simply by the polymer solution with an addition of the Dox at room temperature. To reveal the release behavior of the drug from the hydrogel *in vitro*, the Dox loaded hydrogel was immersed in PBS at a pH of 5.5 or 7.4 over 9 d to record the drug release at regular intervals. From the release curve shown in Figure 2, the drug is released rapidly during the first 24 h with approximate 40% of the initial drug at pH 5.5, as the thermosensitive hydrogel promotes the dispersion of the water-soluble drug with a multipore network structure containing large quantities of water. It presents a similar trend for the water-soluble drug in the other drug-loaded hydrogel (Dong et al., 2016a). Due to the multipore network structure of the thermosensitive hydrogel consisted of PEG-PCL micelles, the drug release from PEG-PCL hydrogel included the Dox diffusion from the hydrogel and PEG-PCL micelles. The release rate decreased obviously after the original quick release. In the next 6 d, the released drug was about 15%, reaching a total release of 55%. In the following days, faint drug release was continued until the ninth day. Because Dox is an acid-sensitive drug, the drug release was slower at a neutral pH than an acid environment. At a neutral pH, the drug release only reached 35% after 9 d with a sustained release. Due to the difference of the microenvironments *in vitro* and *in vivo*, the drug delivery *in vitro* can only reveal the regular of the sustained drug delivery with an intratumoral injection, which can be used as a reference for the evaluation of the drug delivery *in vivo*. In practical use, the drug-loaded hydrogel was injected immediately as soon as possible after preparation based on the simple and fast loading process of the drug-loaded hydrogel, although the drug loaded hydrogel can withstand long-term storage without the spontaneous release of drug contents before the injection administration.

### Fluorescence imaging tracking for the drug release and the hydrogel erosion

**Figure 3.**
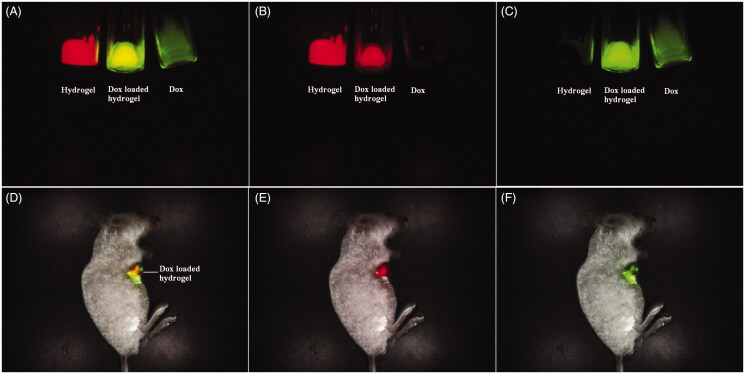
Multispectral fluorescence imaging of mixed fluorescence of the drug and the hydrogel with green and red ((A) Mixed fluorescence of the drug and the hydrogel *in vitro*; (B) Unmixed fluorescence of the hydrogel in vitro, (C) Unmixed fluorescence of the drug *in vitro*; (D) Mixed fluorescence of the drug and the hydrogel *in vivo*; (E)) Unmixed fluorescence of the hydrogel *in vivo*, and (F) Unmixed fluorescence of the drug *in vivo*).

The development of instruments and skills make the fluorescence imaging possible for clinical applications, as the whole-body fluorescence imaging to adult humans has been successfully recorded (Sevick-Muraca, 2012; Piper et al., 2013). The fluorescence imaging for monitoring and tracking will open a new method for accurate drug delivery. For a dual fluorescent drug delivery system, the separation and unmixing of the fluorescent signals of the drug and the carrier is a significant challenge due to the interference of the fluorescent signals. According to the fluorescent difference of the Dox and the porphyrin compound, the multispectral fluorescence imaging recognized successfully the drug and the carrier from the drug-loaded hydrogel. The fluorescence emission signal of the Dox was collected from 560 to 750 nm with the excitation wavelength of 523 nm, while that of the hydrogel was collected from 630 to 800 nm with the excitation wavelength of 595 nm. In view of the difference for each specific fluorescence signal, the difference labels from the drug and the carrier can be distinguished and separated with a multicolor composite image. The *in vitro* fluorescence imaging of the drug-loaded hydrogel was shown in Figure 3(A). A multicolor composite image can distinguish and separate the difference labels from the drug and the carrier with green and red, respectively, because the drug and the carrier emit each specific fluorescent signal by the multispectral analysis spectrum. The Dox loaded hydrogel with yellow stands for the overlap of the Dox with green and the hydrogel with red, signifying the reorganization of the drug and the carrier from the drug delivery system without interference. The satisfactory fluorescence splitting can be applied for the tracking and monitoring of the drug delivery system *in vivo*. When the Dox loaded hydrogel was injected intratumorally into the hepatic tumor, the drug delivery and the tumor location can be visualized by fluorescence imaging and bioluminescence imaging simultaneously. The drug and the carrier still can be separated from the drug-loaded hydrogel by multispectral fluorescence imaging, while the tumor growth or inhibition can be monitored by bioluminescence imaging. The green and red in the tumor area stand for the signal of the DOX and the hydrogel, respectively, while the overlay of their fluorescence signal represented the drug-loaded hydrogel with yellow. The multispectral fluorescence imaging can clearly distinguish the drug and the carrier. The extraction of each single fluorescent signal can monitor and track the material erosion and the drug release by qualitative and quantitatively analysis.

**Figure 4.**
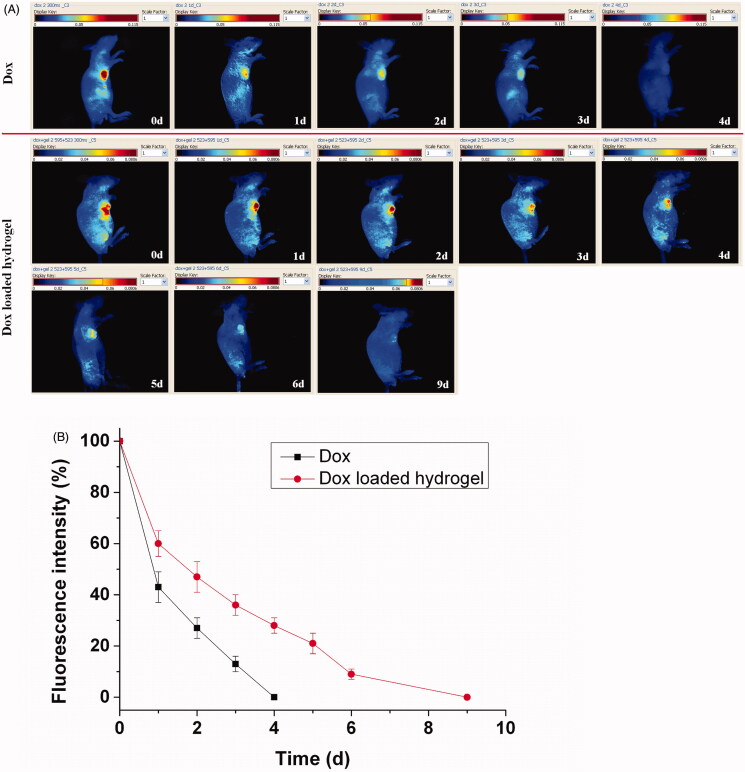
Fluorescence imaging tracking of drug release in vivo after direct Dox injection versus Dox loaded hydrogel injection with rainbow color, with one representative of six in each group (A). Quantative analysis of drug release, the single component of the drug was calculated quantitatively by the Maestro software.The results were expressed as mean ± SD (*n *=6) (B).

The continued imaging analysis for the drug delivery can be shown in Figure 4(A), in which the decay of the fluorescence intensity directly demonstrated the process of the drug release. The fluorescent signal of the Dox from the hydrogel was increasingly reduced for 9 d, because the hydrogel significantly extended the persistent retention of the Dox, while that of the free Dox was rapidly disappeared less than 4 d with the metabolism and distribution of the drug. The imaging results proved the sustained drug release by the entrapment of the hydrogel. A qualitative comparison of the drug release further illustrated the drug delivery process as shown in Figure 4(B). In the drug loaded hydrogel group, a rapid release reached approximate 40% in the first day, and then a steadily sustained decrease was followed for 8 d with 60%. At the ninth day, the disappearance of the fluorescent signal signifies the full drug release. In the free Dox group, an obvious decrease with approximate 60% of the fluorescence happen in the first day and the faint fluorescence was faded away only with 4 d, suggesting the rapid release compared to the Dox loaded hydrogel. However, the *in vivo* results only revealed a reasonable trend and dependence with the *in vitro* release. It caused some difference because of the bioenvironmental factors that affect the diffusion and permeability of the Dox loaded hydrogel *in vitro* and *in vivo*.

**Figure 5.**
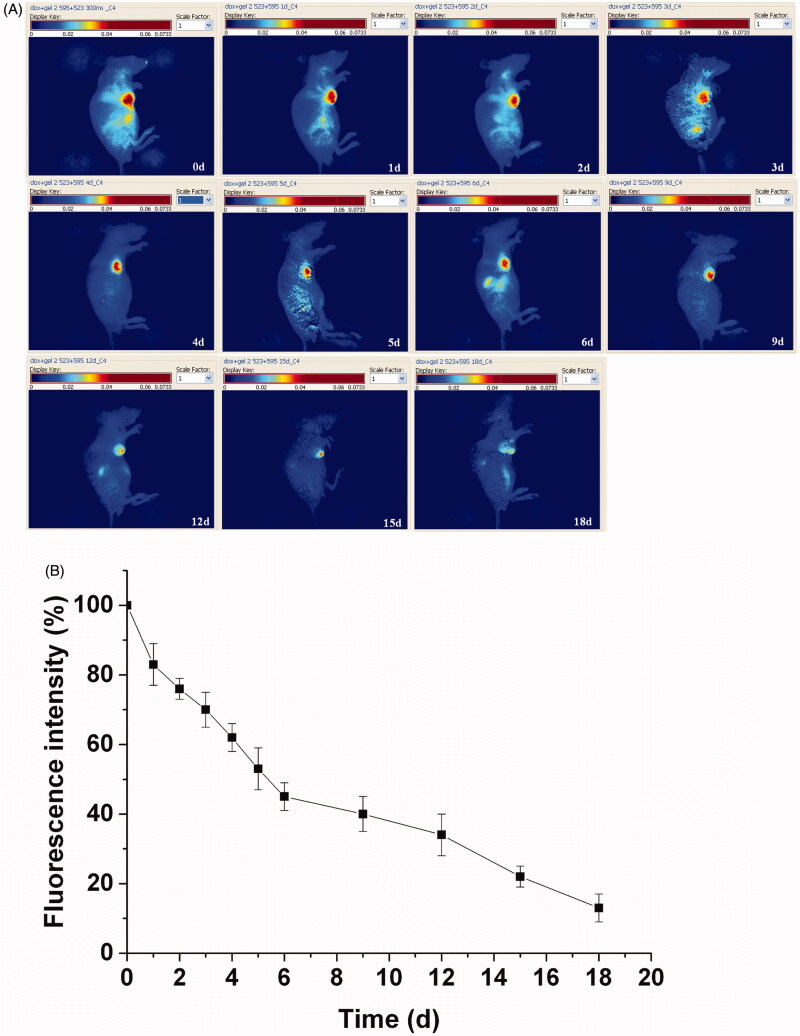
Hydrogel erosion from the hydrogel with rainbow color by fluorescence imaging, with one representative of six in each group. The fluorescence signals of the hydrogel were recorded with an excitation wavelength of 595 nm (A).Quantitative analysis of the hydrogel erosion by fluorescence imaging *in vivo*. The results were expressed as mean ± SD (*n *=6) (B).

As the *in vivo* erosion of the porphyrin incorporated hydrogel has been monitored by subcutaneous implantation before (Dong et al., 2016c), the Dox loaded hydrogel was further tracked for the material erosion by an intratumoral implantation with the potential clinical significance based on the differences between the micro environment of the tumor and the subcutaneous tissue. The delay of the fluorescence signal from the hydrogel was monitored for 18 d to track the detailed *in vivo* erosion process of the hydrogel (Figure 5). A more slow fluorescence decrease of the hydrogel was observed compared to the drug delivery due to the low erosion process of the hydrogel. The fluorescence of the hydrogel shrinked rapidly in the first day with an initial balance of the absorption and permeation. Then a gradual decrease of fluorescence intensities continued with the invasion of the biomolecules. It still remained visible fluorescence even if endured the erosion for 18 d, since the polymer cannot be degraded in a short period. A qualitative analysis about the erosion process of the hydrogel was investigated in Figure 5(B). The fluorescence signal had a decay of 17% within the first day, and then gradually decreases in the next days. After continued erosion over 6 d, it maintained the fluorescence signal of about 45% compared to the original fluorescence. From then on, the hydrogel endowed a sustained erosion to reach a fluorescence of 13% at the 18th day. The hydrogel erosion can be noninvasively monitored from the attenuation of the total fluorescent signals.

Fluorescence imaging is an advanced skill for noninvasive monitoring the drug delivery system at both the cellular and systemic levels (Yokoyama et al., 2012; Wang et al., 2013; Etrych et al., 2016). The fluorescence microscopy techniques are commonly performed for the investigation of the drug delivery, distribution and metabolism at cellular and tissue levels because of the increasing accessibility of abundant fluorescence tags including fluorescence dyes, probes, drugs and proteins (Park et al., 2012; Pampaloni et al., 2013). Their successful applications offer a significant role to reveal the molecular processes of the drug delivery and the diseases therapy in detail. However, fluorescence imaging in whole-body level will face on great difficulty and challenge. Avoiding the scattering effect and the background interference from the bioorganisms is one of the obstacles for the imaging quality, such as the biological chromophore hemoglobin and other biological components from elastin, collagen and other biological fluorophores. Another focus is the imaging sensitivity and accuracy from the fluorescence drug delivery system including the fluorescent drug and fluorescent carrier. Due to the fluorescence interference, the design of a dual fluorescence drug delivery system is a challenging task. Here, a dual fluorescent anti-tumor drug delivery system was designed with a Dox loaded porphyrin incorporated hydrogel base on the fluorescence signals of the Dox and porphyrin. Moreover, fluorescent porphyrin compounds as the backbone and core of the copolymer was incorporated into the hydrogel, overcoming the deficiency of the fluorescent carrier with the exogenous fluorescent tags. Importantly, multispectral fluorescence imaging can successfully separate the fluorescence signals of the drug and the hydrogel from the drug-loaded hydrogel. The single fluorescence of the Dox and the hydrogel could be distinguished with respective signal in the Dox loaded hydrogel. The quantitative analysis of the fluorescence intensities can reveal the drastic process of the drug delivery and the hydrogel erosion.

### *In vivo* bioluminescent imaging for the tumor therapy

**Figure 6.**
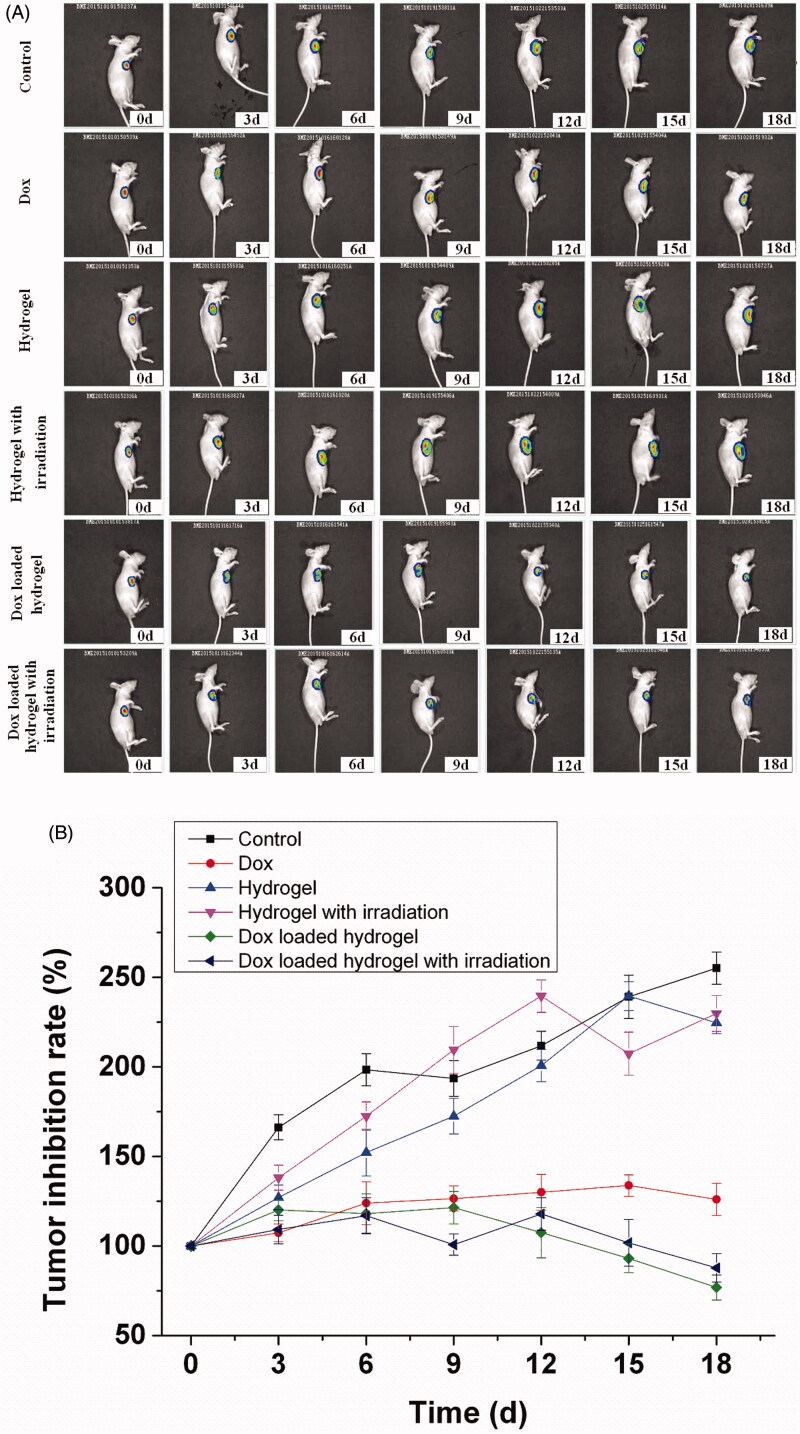
*In vivo* bioluminescence imaging for tumor therapy, with one representative of six in each group. Luciferase expressed hepatic cells Bel-7402 (1 × 10^6^) in 0.1 mL of normal saline (NS) were injected into the armpit region of Balb/c nude mice, which were divided into six different groups including control, Dox, hydrogel, hydrogel with irradiation, Dox loaded hydrogel and Dox loaded hydrogel with irradiation (A). Tumor inhibition rate of drug therapy was recorded after administration from 0 to 18 d by bioluminescence imaging. The results were expressed as mean ± SD (*n* = 6).

Except of the tracking of the drug delivery, the tumor inhibition was monitored with luciferase expression by *in vivo* bioluminescence imaging. Over a period of 18 d following the implantation, the tumor growth or inhibition was depended on the luciferase expression at interval period. As shown in Figure 6(A), bioluminescence imaging revealed a different therapy effect with a dynamic process. The some inhibition of tumor progression can be obvious observed in the free Dox and the Dox loaded hydrogel group, while rapid tumor growth in the control and the hydrogel group. Moreover, the Dox loaded hydrogel inhibited the sustained tumor growth efficiently with a dynamic process. The therapeutic efficacy was further illustrated by the quantitative imaging analysis (Figure 6(B)). The tumor growth was two or three folds after 18 d in the control group and the hydrogel group, while the tumor was inhibited only after administration for 3 d in the Dox loaded hydrogel group and free Dox group. The inhibition effect can continue to the 18th day with inhibition of 30% in the Dox loaded hydrogel group. However, the tumor growth cannot be inhibited further in the free Dox group with the loss of the efficacy due to the rapid metabolism of the Dox. The imaging comparison demonstrated that the Dox loaded hydrogel had longer-lasting anti-tumor effect and therapeutic effect than the free Dox, confirming the sustained release of the drug delivery.

Since porphyrin is a photo sensitive compound, the irradiation effect of the porphyrin incorporated hydrogel is a factor for consideration, which maybe generate some suppressor effect for the tumor under the irradiation damage. In order to investigate the irradiation effect of the porphyrin incorporated hydrogel for the tumor therapy, the tumor inhibition also was tracked under the irradiation with a wavelength of 808 nm each day by bioluminescence imaging. Nevertheless, there was no obvious inhibition effect in the hydrogel group and the Dox loaded hydrogel group under irradiation, which suggested that the porphyrin incorporated hydrogel did not evoke the photodrastic skill effect for the tumor therapy. The main reason maybe comes from the insufficient content and the structural change of the photosensitive compound. It is only our speculation and the detailed investigation need to be revealed in the following study.

Overall, the optical imaging can visualize the whole process of the drug delivery and the tumor therapy using nude mice bearing luciferase expressed hepatic tumor as models. The dual fluorescent drug delivery system was monitored *in vivo* by multispectral fluorescence imaging system, while the tumor growth and inhibition effects was tracked by bioluminescence imaging along with the continued release of the drug. The fluorescence imaging and bioluminescence imaging were complementary imaging skills without mutual interference for imaging guiding. Moreover, the interrelation between the drug delivery and the tumor therapy were illustrated based on the imaging location and tracking. The image guiding for the drug delivery and treatment can explore a potential direction for medical imaging, affording the possibility of the precise drug delivery and treatment.

## Conclusion

In summary, a dual fluorescent drug delivery system consisted of the Dox loaded POR-PEG-PCL hydrogel was successfully designed for the tracking of the drug delivery and the tumor therapy by fluorescence imaging and bioluminescence imaging. The dual fluorescent drug delivery system was monitored *in vivo* by multispectral fluorescence imaging system, while the tumor growth and inhibition effects was tracked by bioluminescence imaging along with the continued release of the drug. The imaging comparison demonstrated that the Dox loaded hydrogel had longer-lasting anti-tumor effect and therapeutic effect than the free Dox, confirming the sustained release of the drug delivery. The image guiding for the drug delivery and treatment can explore a potential direction for medical imaging, affording the possibility of the precise drug delivery and treatment.

## References

[CIT0001] Ahmed Abdelbary A, Elsayed I, Hassen Elshafeey A. (2015). Design and development of novel lipid based gastroretentive delivery system: response surface analysis, *in-vivo* imaging and pharmacokinetic study. Drug Deliv 22:37–49. 24350634 10.3109/10717544.2013.868960

[CIT0002] Appel AA, Anastasio MA, Larson JC, Brey EM. (2013). Imaging challenges in biomaterials and tissue engineering. Biomaterials 34:6615–30. 23768903 10.1016/j.biomaterials.2013.05.033 PMC3799904

[CIT0003] Brudno Y, Silva EA, Kearney CJ, et al. (2014). Refilling drug delivery depots through the blood. Proc Natl Acad Sci USA 111:12722–7. 25139997 10.1073/pnas.1413027111 PMC4156738

[CIT0004] Bruggeman JP, De Bruin BJ, Bettinger CJ, Langer R. (2008). Biodegradable poly(polyol sebacate) polymers. Biomaterials 29:4726–35. 18824260 10.1016/j.biomaterials.2008.08.037 PMC2948970

[CIT0005] Chen D, Dougherty CA, Zhu K, Hong H. (2015). Theranostic applications of carbon nanomaterials in cancer: focus on imaging and cargo delivery. J Control Release 210:230–45. 25910580 10.1016/j.jconrel.2015.04.021

[CIT0006] Cunha-Reis C, El Haj AJ, Yang X, Yang Y. (2013). Fluorescent labeling of chitosan for use in non-invasive monitoring of degradation in tissue engineering. J Tissue Eng Regen Med 7:39–50. 22125289 10.1002/term.494

[CIT0007] Dong X, Sun Z, Wang X, et al. (2017). Simultaneous monitoring of the drug release and antitumor effect of a novel drug delivery system-Mwcnts/Dox/Tc. Drug Deliv 24:143–51. 28156171 10.1080/10717544.2016.1233592 PMC8241058

[CIT0008] Dong X, Wei C, Chen H, et al. (2016a). Real-time imaging tracking of a dual fluorescent drug delivery system based on zinc phthalocyanine-incorporated hydrogel. ACS Biomater Sci Eng 2:2001–10. 33440536 10.1021/acsbiomaterials.6b00403

[CIT0009] Dong X, Wei C, Liu T, et al. (2016b). Real-time fluorescence tracking of protoporphyrin incorporated thermosensitive hydrogel and its drug release *in vivo*. ACS Appl Mater Interfaces 8:5104–13. 26848506 10.1021/acsami.5b11493

[CIT0010] Dong X, Wei C, Lu L, et al. (2016c). Fluorescent nanogel based on four-arm PEG-PCL copolymer with porphyrin core for bioimaging. Mater Sci Eng C Mater Biol Appl 61:214–19. 26838843 10.1016/j.msec.2015.12.037

[CIT0011] Dong X, Wei C, Liu T, Lv F. (2015). Protoporphyrin incorporated alginate hydrogel: preparation, characterization and fluorescence imaging *in vivo*. RSC Adv 117:96336–44.

[CIT0012] Etrych T, Lucas H, Janouskova O, et al. (2016). Fluorescence optical imaging in anticancer drug delivery. J Control Release 226:168–81. 26892751 10.1016/j.jconrel.2016.02.022

[CIT0013] Ghaderi S, Ramesh B, Seifalian AM. (2011). Fluorescence nanoparticles “quantum dots” as drug delivery system and their toxicity: a review. J Drug Target 19:475–86. 20964619 10.3109/1061186X.2010.526227

[CIT0014] Greenaway C, Ratnaraj N, Sander JW, Patsalos PN. (2010). A high-performance liquid chromatography assay to monitor the new antiepileptic drug lacosamide in patients with epilepsy. Ther Drug Monit 32:448–52. 20386357 10.1097/FTD.0b013e3181dcc5fb

[CIT0015] Hilderbrand SA, Weissleder R. (2010). Near-infrared fluorescence: application to *in vivo* molecular imaging. Curr Opin Chem Biol 14:71–9. 19879798 10.1016/j.cbpa.2009.09.029

[CIT0016] Hoffmann S, Vystrcilova L, Ulbrich K, et al. (2012). Dual fluorescent HPMA copolymers for passive tumor targeting with pH-sensitive drug release: synthesis and characterization of distribution and tumor accumulation in mice by noninvasive multispectral optical imaging. Biomacromolecules 13:652–63. 22263698 10.1021/bm2015027

[CIT0017] Huang H, Hernandez R, Geng J, et al. (2016). A porphyrin-PEG polymer with rapid renal clearance. Biomaterials 76:25–32. 26517562 10.1016/j.biomaterials.2015.10.049 PMC4662896

[CIT0018] Kruger HR, Schutz I, Justies A, et al. (2014). Imaging of doxorubicin release from theranostic macromolecular prodrugs via fluorescence resonance energy transfer. J Control Release 194:189–96. 25176577 10.1016/j.jconrel.2014.08.018

[CIT0019] Li D, Zhang YT, Yu M, et al. (2013). Cancer therapy and fluorescence imaging using the active release of doxorubicin from MSPs/Ni-LDH folate targeting nanoparticles. Biomaterials 34:7913–22. 23886730 10.1016/j.biomaterials.2013.06.046

[CIT0020] Liang SL, Yang XY, Fang XY, et al. (2011). *In vitro* enzymatic degradation of poly (glycerol sebacate)-based materials. Biomaterials 32:8486–96. 21855132 10.1016/j.biomaterials.2011.07.080

[CIT0021] Lin Q, Huang H, Chen J, Zheng G. (2016). Using fluorescence imaging to track drug delivery and guide treatment planning *in vivo*. Methods Mol Biol 1444:153–66. 27283425 10.1007/978-1-4939-3721-9_14

[CIT0022] Lv F, Mao L, Liu T. (2014). Thermosensitive porphyrin-incorporated hydrogel with four-arm PEG-PCL copolymer: preparation, characterization and fluorescence imaging *in vivo*. Mater Sci Eng C Mater Biol Appl 43:221–30. 25175208 10.1016/j.msec.2014.07.019

[CIT0023] Ma X, Hui H, Shang W, et al. (2015). Recent advances in optical molecular imaging and its applications in targeted drug delivery. Curr Drug Targets 16:542–8. 25557258 10.2174/1389450116666150102112747

[CIT0024] Pampaloni F, Ansari N, Stelzer EH. (2013). High-resolution deep imaging of live cellular spheroids with light-sheet-based fluorescence microscopy. Cell Tissue Res 352:161–77. 23443300 10.1007/s00441-013-1589-7

[CIT0025] Park CW, Rhee YS, Vogt FG, et al. (2012). Advances in microscopy and complementary imaging techniques to assess the fate of drugs *ex vivo* in respiratory drug delivery: an invited paper. Adv Drug Deliv Rev 64:344–56. 21920394 10.1016/j.addr.2011.08.004

[CIT0026] Piper SK, Habermehl C, Schmitz CH, et al. (2013). Towards whole-body fluorescence imaging in humans. PLoS One 8:e83749. 24391820 10.1371/journal.pone.0083749 PMC3877082

[CIT0027] Rieffel J, Chen F, Kim J, et al. (2015). Hexamodal imaging with porphyrin-phospholipid-coated upconversion nanoparticles. Adv Mater 27:1785–90. 25640213 10.1002/adma.201404739 PMC4416944

[CIT0028] Selvam S, Kundu K, Templeman KL, et al. (2011). Minimally invasive, longitudinal monitoring of biomaterial-associated inflammation by fluorescence imaging. Biomaterials 32:7785–92. 21813173 10.1016/j.biomaterials.2011.07.020 PMC3159805

[CIT0029] Sevick-Muraca EM. (2012). Translation of near-infrared fluorescence imaging technologies: emerging clinical applications. Annu Rev Med 63:217–31. 22034868 10.1146/annurev-med-070910-083323

[CIT0030] Song F, Li X, Wang Q, et al. (2015). Nanocomposite hydrogels and their applications in drug delivery and tissue engineering. J Biomed Nanotechnol 11:40–52. 26301299 10.1166/jbn.2015.1962

[CIT0031] Tzu-Yin W, Wilson KE, Machtaler S, Willmann JK. (2013). Ultrasound and microbubble guided drug delivery: mechanistic understanding and clinical implications. Curr Pharm Biotechnol 14:743–52. 24372231 10.2174/1389201014666131226114611 PMC4084724

[CIT0032] Undin J, Finne-Wistrand A, Albertsson AC. (2014). Adjustable degradation properties and biocompatibility of amorphous and functional poly(ester-acrylate)-based materials. Biomacromolecules 15:2800–7. 24915542 10.1021/bm500689g

[CIT0033] Wang K, He X, Yang X, Shi H. (2013). Functionalized silica nanoparticles: a platform for fluorescence imaging at the cell and small animal levels. Acc Chem Res 46:1367–76. 23489227 10.1021/ar3001525

[CIT0034] Wang W, Liu J, Li C, et al. (2014). Real-time and non-invasive fluorescence tracking of *in vivo* degradation of the thermosensitive PEGlayted polyester hydrogel. J Mater Chem B 2:4185–92. 32261752 10.1039/c4tb00275j

[CIT0035] Winzen S, Koynov K, Landfester K, Mohr K. (2016). Fluorescence labels may significantly affect the protein adsorption on hydrophilic nanomaterials. Coll Surf B 147:124–8. 10.1016/j.colsurfb.2016.07.057 27497932

[CIT0036] Wohl-Bruhn S, Badar M, Bertz A, et al. (2012). Comparison of *in vitro* and *in vivo* protein release from hydrogel systems. J Control Release 162:127–33. 22687287 10.1016/j.jconrel.2012.05.049

[CIT0037] Woods A, Patel A, Spina D, et al. (2015). *In vivo* biocompatibility, clearance, and biodistribution of albumin vehicles for pulmonary drug delivery. J Control Release 210:1–9. 25980621 10.1016/j.jconrel.2015.05.269 PMC4674532

[CIT0038] Xia Y, Matham MV, Su H, et al. (2016). Nanoparticulate contrast agents for multimodality molecular imaging. J Biomed Nanotechnol 12:1553–84. 29341579 10.1166/jbn.2016.2258

[CIT0039] Yokoyama N, Otani T, Hashidate H, et al. (2012). Real-time detection of hepatic micrometastases from pancreatic cancer by intraoperative fluorescence imaging: preliminary results of a prospective study. Cancer 118:2813–19. 21990070 10.1002/cncr.26594

[CIT0040] Zhang Q, Mochalin VN, Neitzel I, et al. (2011). Fluorescent PLLA-nanodiamond composites for bone tissue engineering. Biomaterials 32:87–94. 20869765 10.1016/j.biomaterials.2010.08.090

[CIT0041] Zhang Y, Rossi F, Papa S, et al. (2016). Non-invasive *in vitro* and *in vivo* monitoring of degradation of fluorescently labeled hyaluronan hydrogels for tissue engineering applications. Acta Biomater 30:188–98. 26621694 10.1016/j.actbio.2015.11.053

[CIT0042] Zhang Y, Yang J. (2013). Design strategies for fluorescent biodegradable polymeric biomaterials. J Mater Chem B Mater Biol Med 1:132–48. 23710326 10.1039/C2TB00071G PMC3660738

[CIT0043] Zhou H, Hernandez C, Goss M, et al. (2015). Biomedical imaging in implantable drug delivery systems. Curr Drug Targets 16:672–82. 25418857 10.2174/1389450115666141122211920 PMC4441594

[CIT0044] Zhou L, El-Deiry WS. (2009). Multispectral fluorescence imaging. J Nucl Med 50:1563–6. 19759119 10.2967/jnumed.109.063925

[CIT0045] Zhu D, Chen Z, Zhao K, et al. (2015). Polypropylene non-woven supported fibronectin molecular imprinted calcium alginate/polyacrylamide hydrogel film for cell adhesion. Chin Chem Lett 26:807–10.

[CIT0046] Zhu G, Zhang Y, Wang K, et al. (2016). Visualized intravesical floating hydrogel encapsulating vaporized perfluoropentane for controlled drug release. Drug Deliv 23:2820–6. 26515239 10.3109/10717544.2015.1101791

